# Impact of music on pain perception during office-based transperineal prostate biopsy: a prospective non-randomized study

**DOI:** 10.1038/s41598-026-41323-7

**Published:** 2026-04-24

**Authors:** Luca Montrone, Marco Finati, Anna Ricapito, Antonio Fanelli, Nicolò Giannoccaro, Francesco Troiano, Giuliano Ciavotta, Ugo Giovanni Falagario, Luigi Cormio, Gian Maria Busetto, Carlo Bettocchi, Oscar Selvaggio, Giuseppe Carrieri

**Affiliations:** 1 Department of Urology and Organ Transplantation, University of Foggia, Foggia, Italy; 2 Department of Urology, Ospedale L. Bonomo, Andria, Italy; 3 Department of Urology and Renal Transplantation, University of Foggia, Viale Luigi Pinto 1, Foggia, 71122 FG Italy

**Keywords:** Transperineal prostate biopsy, Music therapy, Pain management, Office-based procedures, Visual analog scale, Non-pharmacological interventions, Health care, Medical research, Neuroscience

## Abstract

Although music has been shown to alleviate pain and anxiety during transrectal PBx, limited evidence is available regarding its impact in the transperineal (TP) setting. This study aimed to investigate the effect of music on pain perception during office-based TP PBx. This prospective, comparative, non-randomized study enrolled biopsy-naïve patients undergoing TP PBx between March 2024 and March 2025. A total of 200 patients were equally assigned to the intervention group (listening to music during PBx) or the control group. Pain was assessed using the Visual Analog Scale (VAS) at six predefined timepoints (T0–T5). A differential pain score (D-VAS) was calculated at each timepoint, using the VAS score at T0 as the baseline. Changes over time between the two groups were analyzed using independent t-tests and a linear mixed-effects model. An interaction term between music and timepoints was included to test whether the effect of music on pain perception varied across the different stages of the procedure. Compared with controls, D-VAS was significantly lower in the music group at the first core (T3, *p* = 0.025), last core (T4, *p* < 0.001) and at discharge (T5, *p* < 0.001). No significant differences were observed during local anesthesia (T1-T2). The Mixed-effects model confirmed these findings and revealed that both pain perception and the relative analgesic effect of music varied accross different stages of the procedure. Listening to music was associated with lower pain perception during TP PBx, particularly during biopsy sampling phases, and resulted in lower pain levels at discharge.

**Supplementary Information:**

The online version contains supplementary material available at 10.1038/s41598-026-41323-7.

## Introduction

The European Association of Urology (EAU) guidelines recommend the transperineal (TP) approach as the preferred method for prostate biopsy (PBx), given its significantly lower risk of infectious complications compared to the transrectal (TR) route^1^. This evidence originates from previous studies that have demonstrated not only a lower risk of sepsis and urinary tract infections with the TP approach^2^, but also better access to the anterior and apical regions of the prostate, which are often undersampled^3,4^.

That said, TP PBx is still associated with greater perceived pain compared to the TR approach, despite adequate sedation or anesthesia^5^. This factor not only negatively affect the patient’s experience and their willingness to undergo repeat procedures, but also limit the adoption of TP PBx as a routine office-based practice^6^.

Music has been recognized as an effective non-pharmacological intervention for reducing pain, anxiety, and stress in various clinical settings. Listening to music during medical procedures has shown significant benefits, including reduced perception of pain and anxiety, and decreased need for analgesics^7,8^.

In the field of urology, listening to music during procedures such as flexible cystoscopy has led to significant reductions in pain and anxiety^9^, suggesting the potential utility of this intervention in similar contexts, such as PBx. Chang et al. reported that the use of music during TR PBx exerted a calming effect, resulting in a significant reduction in both mean anxiety levels and mean pain scores, compared with patients who did not receive music^10^. Another randomized controlled trial demonstrated that the addition of music during fusion TR PBx, alongside standard pain management, significantly reduced pain and anxiety levels^11^. Given its simplicity, cost-effectiveness and non-invasive nature, music represents a valuable adjunct for routine use in this kind of procedures to enhance pain palliation.

To our knowledge, no studies have specifically investigated the role of music in modulating pain perception during TP PBx.

This study aimed to evaluate the effect of music on pain perception during office-based TP PBx and to explore how this effect varied across different stages of the procedure. We hypothesized that music would significantly attenuate pain perception, particularly during the core sampling phase of the procedure.

## Materials and methods

### Study design and population

This is a prospective, comparative, non-randomized study enrolling biopsy-naïve patients who underwent TP PBx in an outpatient setting at an academic Institution, between March 2024 and March 2025.

Exclusion criteria included: previous PBx, current therapy with antidepressants, antipsychotics, or analgesics, previous diagnosis of diabetes mellitus, presence of an indwelling urinary catheter, hearing impairment. Patients were divided into two groups:

- Group A (intervention): TP PBx with music played during the procedure.
- Group B (control): TP PBx without additional interventions.

Patients signed an informed consent prior the procedure and then were randomly assigned to a group. Patients who did not complete the procedure due to excessive discomfort or pain were excluded from the analysis.

### Anesthesia, biopsy protocol and intervention

Antibiotic prophylaxis and rectal cleansing were routinely performed from the night before the procedure. A topical anesthetic cream containing lidocaine-prilocaine (LP) was applied to the perianal and intrarectal (PI) regions 60 min before the procedure, at the bedside of the patient’s room, and VAS questionnaire was administered. The patient was then moved to the office operating room, where PBx is performed. The patient was placed in the lithotomy position, and the room was closed for the duration of the procedure, with only two surgeons and a nurse present. At this point, patients in the intervention group were allowed to select their preferred music genre, and the volume was adjusted according to their preference. Music was played from a portable Bluetooth device placed behind patient’s head, beginning a few minutes before subcutaneous anesthesia and continuing until the end of the procedure. Local anesthesia was then administered using a total of 20 mL of 2% lidocaine, distributed for subcutaneous and periprostatic nerve blocks. Systematic biopsy was performed using a bkFusion™ ultrasound machine, following a 12-cores standard mapping free-hand technique under TR ultrasound guidance. When MRI showed a suspicious lesion (PIRADS > 2) Targeted PBx was performed using Koelis™ Trinity machine. In such cases, a TR ultrasound scan was obtained after subcutaneous anesthesia, and the VAS scale was administered. The probe was temporarily removed from the rectum while contouring the lesion and the prostate. Three to five additional cores were taken for each suspicious lesion. Patients who did not complete the standard systematic template were excluded from the analysis, regardless of whether targeted PBx was also performed.

### Outcomes measure

Perceived pain was assessed using the Visual Analog Scale (VAS), a validated tool ranging from 0 (“no pain”) to 10 (“worst imaginable pain”). The VAS is widely employed in acute pain evaluation during invasive procedures due to its simplicity, sensitivity, and reproducibility^12,13^.

VAS scores were registered at six specific time points:

1. T0: After digital rectal examination and perianal-intrarectal lidocaine-prilocaine (PI-LP) cream application. This baseline pain assessment was performed in the patient’s hospitalization room prior to transfer to the procedure room, and therefore before any exposure to the music intervention, which was initiated only after patient positioning in the operating room and shortly before subcutaneous anesthesia.
2. T1: After subcutaneous anesthesia.
3. T2: Periprostatic block.
4. T3: After first biopsy core.
5. T4: Last biopsy core.
6. T5: At discharge.

The following demographical and clinical variables were also recorded: Age of patients, prostate volume (cc), number of cores taken and type of biopsy (targeted + systematic versus systematic only).

### Statistical and power analysis

Pairwise pain comparisons at individual timepoints using Mann-Whitney-U test were performed as descriptive analyses to illustrate between-group differences at specific procedural stages. To control for initial pain perception, a differential pain score (D-VAS) was calculated at each timepoints, using the VAS score at T0 as the baseline (D-VAS = VAS_Tn – VAS_T0). This approach was chosen to capture the time-varying nature of pain across sequential biopsy stages and to account for baseline differences in pain perception observed before the initiation of the music intervention. Change in D-VAS from baseline at each timepoint were then compared, and sensitivity analyses based on type of biopsy (targeted + systematic versus systematic only) were also performed.

To evaluate the effect of music on pain perception throughout the prostate biopsy procedure, we constructed a linear mixed-effects model with the change in pain score from baseline (D-VAS) as the dependent variable. The model included a categorical time variable representing each predefined procedural timepoint (T1 to T5), at which D-VAS scores were recorded. The model was adjusted for age, number of biopsy cores, biopsy type (systematic only vs. targeted plus systematic) and baseline VAS (at T0). An interaction term between group (music vs. control) and time was then included to test whether the impact of the music intervention on pain perception varied across the different stages of the procedure. A random intercept for each patient was included to model within-subject correlation arising from repeated pain assessments, while estimating population-level effects of time and intervention. The model was estimated using restricted maximum likelihood (REML), as recommended for longitudinal data with repeated measures^14^.Model diagnostics were performed as post-estimation checks, and visual inspection of residual and Q–Q plots did not indicate substantial departures from normality or homoscedasticity. For illustrative purpose, the predicted D-VAS were graphically displayed at each timepoints for Music vs. Control group.

All analysis were performed using RStudio© (Rstudio Team, 2024.12.1.563). A significance threshold of *p* < 0.05 was used for all tests, with statistically significant values reported in bold. Descriptive comparisons of pain scores between groups at individual timepoints are reported for clinical interpretability; however, inferential conclusions are based on the mixed-effects model.

Based on prior studies on music intervention during PBx, we assumed a clinically meaningful difference of 1.0 point on the D-VAS scale, with a standard deviation (SD) of 2.5, corresponding to a Cohen’s d of 0.4. To detect this effect with 80% power and a two-sided alpha of 0.05, a total of 200 participants (100 per group) is required. This sample size also provides sufficient power for the mixed-model longitudinal analysis, assuming moderate correlations (C ≈ 0.5) among repeated measures.

## Results

Three patients did not complete the procedure due to excessive pain/discomfort: 2 in the control group for symptomatic hemorrhoids, and 1 in the music group who previously underwent rectal surgery. These patients were excluded from analysis and their baseline characterisctics are reported in **Supplementary Table 1.** The final cohort included 200 patients equally distributed in the two (Control vs. Music) groups. Baseline demographic and clinical characteristics (reported in Table 1) were homogeneous between the two groups, except for a higher rate of patients undergoing fusion + systematic PBx in the control group (52% vs. 11%).

**Table 1:** Clinical and demographical variables of the cohort.

	Control Group (*N* = 100)	Music Group (*N* = 100)	*p* value*
Age, years Median (Q1, Q3)	67 (62, 71)	65 (60, 72)	0.2
**Type of Biopsy**,** n (%)** Systematic Only Systematic+Targeted	48 (48.0%) 52 (52.0%)	89 (89.0%) 11 (11.0%)	**< 0.001**
**Prostate cores** Median (Q1, Q3)	12 (12–13)	12 (12, 12)	0.5
**Prostate volume**,** cc** Median (Q1, Q3)	50 (40, 70)	50 (39, 78)	0.2
**Visual Analog Scale (VAS) score**			
**T0: PI-LP application** Mean (SD)	1.24 (1.44)	1.94 (1.19)	**< 0.001**
**T1: Subcutaneous anesthesia** Mean (SD)	1.99 (1.42)	2.64 (1.18)	**< 0.001**
**T2: Periprostatick Block** Mean (SD)	3.13 (2.03)	3.39 (1.61)	0.3
**T3: First Biopsy Core** Mean (SD)	3.04 (2.35)	2.98 (1.66)	0.8
**T4: Last Biopsy Core** Mean (SD)	2.70 (1.96)	2.08 (1.25)	**0.008**
**T5: Discharge** Mean (SD)	0.97 (0.99)	0.98 (0.83)	0.9

PI-LP: lidocaine-prilocaine.

*from chi-square or Mann-Whitney U test, as appropriate.

The overall mean VAS (SD) score across all procedural phases was 2.13 (0.98) in the music group and 2.38 (1.05) in the control group (*p* = 0.09). At T0 (digital rectal examination + PI-LP application), patients in the music group reported significantly higher pain scores compared to the control group (mean VAS: 1.94 vs. 1.24, *p* < 0.001). This difference remained after local anesthesia (T1, mean: 2.64 vs. 1.99, *p* = 0.001), while no statistically significant differences in absolute pain scores were observed at periprostatic block (T2) and at time of first biopsy core (T3). At last core (T4), VAS score was significantly lower in the music group (mean: 2.08 vs. 2.70; *p* = 0.008), while no difference was observed at discharge (T5, see Table 1).

### Changes from baseline (D-VAS)

Table 2 reported the change in VAS score from baseline (T0) to each timepoint. There were no significant differences in D-VAS between groups at T1 and T2. However, at T3 (first core), the music group reported a significantly smaller increase in pain (mean D-VAS: 1.04 vs. 1.80; *p* = 0.025), and this difference was more pronounced at last core (mean D-VAS at T4: 0.14 vs. 1.46; *p* < 0.001). At discharge (T5), the music group showed a significantly greater reduction in pain relative to baseline (-0.96 vs. -0.27; *p* < 0.001).

**Table 2:** Differential Pain Score from baseline Visual Analog Scale score (D-VAS) at each timepoints for Music and Control Group.

	Control Group (*N* = 100)	Music Group (*N* = 100)	Mean Difference (Music – Control), 95% CI	*p* value*
D-VAS: T1-T0 Mean (SD)	0.75 (1.31)	0.70 (1.07)	−0.05 (− 0.41; 0.31)	0.8
D-VAS: T2-T0 Mean (SD)	1.89 (2.49)	1.45 (1.55)	−0.44 (− 1.02; 0.14)	0.6
**D-VAS: T3-T0** Mean (SD)	1.80 (2.87)	1.04 (1.76)	−0.76 (− 1.42; −0.10)	**0.033**
**D-VAS: T4-T0** Mean (SD)	1.46 (2.50)	0.14 (1.30)	−1.32 (− 1.85; −0.79)	**< 0.001**
**D-VAS: T5-T0** Mean (SD)	-0.27 (1.43)	-0.96 (1.26)	−0.69 (− 1.09; −0.29)	**< 0.001**

*from Mann-Whitney U test.

### Subgroup analysis

Among patients undergoing systematic biopsy only, the music group initially reported higher pain at T0 (2.00 vs. 1.21; *p* = 0.002) and T1 (2.64 vs. 2.00; *p* = 0.007). However, this reversed over the course of the procedure, and by T4, music group reported lower pain scores (2.04 vs. 2.69; *p* = 0.05). At first biopsy core (T3), the music group showed a significantly lower increase in pain compared to controls (mean D_VAS: 0.91 vs. 2.19, *p* = 0.01). This difference was even more pronounced at last biopsy core (mean D-VAS: 0.045 vs. 1.48, *p* = 0.001) and remained significant at discharge (T5: − 1.01 vs. − 0.19, *p* = 0.001).

### Mixed-effects model (Table 3)

**Table 3:** Linear Mixed-Effects model estimating D-VAS, An interaction term was introduced to test the impact of Music Intervention on D-VAS at each timepoints.

Covariates	Estimate	95% CI Lower	95% CI Upper	*P*-value
Age	-0,001	-0,016	0,015	0.9
VAS at T0	0.24	0.13	0.35	**< 0.001**
T2 vs. T1	1,14	0,78	1,50	**< 0.001**
T3 vs. T2	1,05	0,69	1,41	**< 0.001**
T4 vs. T3	0,71	0,35	1,07	**< 0.001**
T5 vs. T4	-1,02	-1,38	-0,66	**< 0.001**
Nr of Biopsy cores	0,043	-0,041	0,13	0.3
Target + Systematic PBx vs. Syst. only	-0,093	-0,44	0,25	0.6
T1: Music vs. Group	0.44	-0,029	0,89	0.067
T2: Music vs. Control	-0,39	-0,90	0,12	0.1
T3: Music vs. Control	-0,71	-1,22	-0,20	**0.007**
T4: Music vs. Control	-1,27	-1,78	-0,76	**< 0.001**
T5: Music vs. Control	-0,64	-1,15	-0,13	**0.015**

**Fig. 1:**
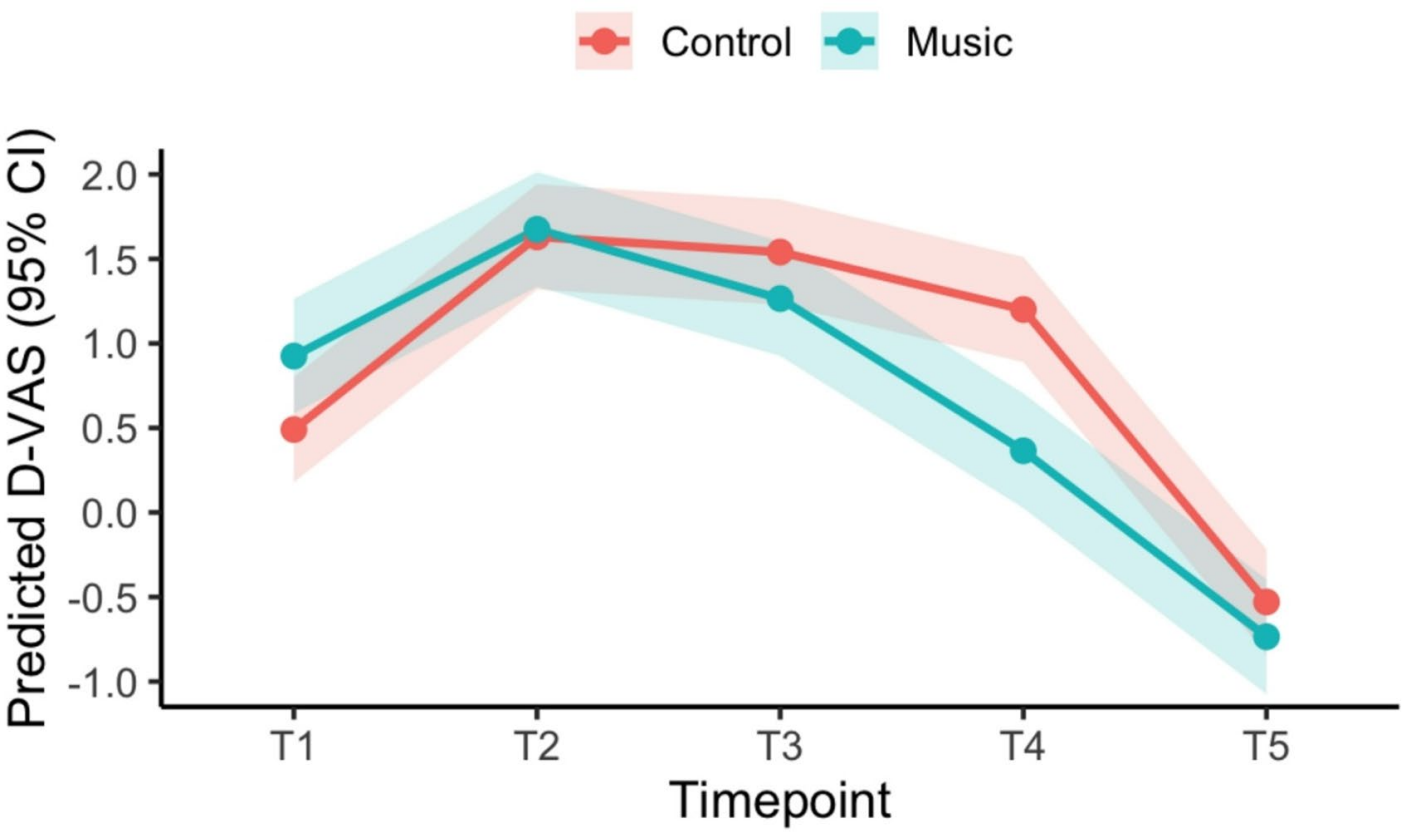
Predicted D-VAS at each Timepoints for Control and Music Group.

The model revealed a significant main effect of time (*p* < 0.001), reflecting a rise in pain during biopsy (T2-T4) and decline after the procedure (T5). Importantly, baseline pain intensity (VAS at T0) was independently associated with higher pain scores throughout the procedure (β = 0.24 p, 95% CI 0.13–0.35; *p* < 0.001), The interaction term was significant, revealing that music was associated with reduced pain, when compared to control at: T3 (first core): -0.71 (95% CI: -1.22 to -0.20; *p* = 0.007), T4 (last core): -1.27 (95% CI: -1.78 to -0.76; *p* < 0.001) and T5 (discharge): -0.64 (95% CI: -1.15 to -0.13; *p* = 0.015). No significant difference between groups was observed at early procedural stages (T1–T2) and none of the other covariates were independently associated with D-VAS. The predicted D-VAS at each timepoint for music and control group are displayed in Fig. 1. The mixed-effects model was then repeated in a matched cohort of patients equally receiving either systematic + fusion versus systematic-only PBx (see **Supplementary Table 2**), revealing a trend consistent with the primary analysis.

## Discussion

Due to its lower risk of infection and superior sampling of the anterior prostate, TP PBx is the first recommended approach for prostate tissue sampling, as recommended by the EAU PBx guidelines^1,3^. Despite these advantages, pain perception remains a major barrier to its widespread implementation, particularly in office-based settings where the anesthesiologist support is often not available^6^. As published by Bagheri et al., music therapy is a non-pharmacological and non-invasive mind–body intervention that influences central nervous system processing, modulates pain perception, reduces sympathetic nervous system activation, and promotes emotional regulation. Its clinical effectiveness has been demonstrated across a variety of surgical settings, with consistent evidence supporting its role in reducing postoperative pain and anxiety^15^. Music has previously been shown to reduce pain and anxiety during various urological procedures, including cystoscopy and TR PBx^9,11^. However, evidence supporting its effectiveness for TP PBx is limited. This prospective non-randomized study aimed to assess whether music intervention during office-based TP PBx can significantly reduce perceived pain. Our findings are severalfold. First, although patients in the music group experienced higher pain at digito-rectal examination and subcutaneous anesthesia, no difference in pain levels was observed during core sampling phase and at discharge. Second, because pain perception is inherently subjective and highly variable between individuals, we adjusted for baseline pain using VAS at T0. This early pain score following DRE and LP-IP application served as a proxy for each patient’s baseline discomfort threshold, helping to control for individual variability before music intervention was started. After this adjustment, the analysis revealed that the analgesic effect of music was stage-dependent and most pronounced during biopsy sampling and at discharge, compared to controls. That said, baseline pain intensity (VAS at T0) was independently associated with higher pain scores throughout the procedure, indicating substantial inter-individual variability in pain sensitivity. Third, the music intervention proved to be effective in the subgroup undergoing systematic biopsy only, while limited sample size precluded sensitivity analyses in other subgroups; larger studies will be required to determine whether longer or more complex procedures, such as fusion PBx, modify the impact of music on pain modulation. Fourth, we constructed a linear mixed-effects model using change in pain from baseline as the outcome to evaluate how pain evolved throughout the procedure and whether music influenced this pattern – rather than assuming a uniform effect. This approach allowed us to account for within-subject correlation across timepoints and to adjust for potential confounders such as age, number of biopsy cores, and biopsy type. From a clinical perspective, our findings suggest that music may emerge as a stage-specific analgesic adjunct, with its impact becoming apparent during biopsy sampling after longer exposure. Even modest reductions in pain at these stages may translate into improved procedural tolerance, reduced distress, and a more positive overall patient experience. Given the simplicity, safety, and low cost of the intervention, these findings support the potential role of music as a feasible adjunct to standard analgesic protocols during TP PBx, particularly during tissue sampling.

Music is known to exert its analgesic effects through multiple mechanisms, including distraction, emotional engagement, and modulation of the limbic and autonomic nervous systems^16^. Its use has been associated with reductions in anxiety, heart rate, blood pressure, and opioid requirement in various clinical settings^17^. In urology, Mumm et al. demonstrated similar benefits during flexible cystoscopy, with significant reductions in patient-reported pain and anxiety scores^9^.Previous randomized controlled studies confirmed its efficacy during TR PBx, even during latest MRI-targeted procedures^10,11,18^. For instance, Baba et al. demonstrated that baseline discomfort, patient preparation, and anxiety levels substantially shape patient-reported outcomes during TRUS biopsy, underscoring the importance of accounting for pre-intervention pain and psychological state^19^. Compared with TR PBx, the TP approach differs in patient positioning, access route, and anesthetic strategy, while fusion-guided procedures in both settings introduce additional that prolongs duration and cognitive workload. In the absence of randomized trial in the TP setting, a recent pilot study by Öztürk et al. explored the effect of music during TP PBx on a limited sample size of 97 patients, by assessing pain at three (probe insertion, local anesthesia and biopsy shot)^20^. Although still not randomized, our study strengthens and extends this evidence through a more robust design, using a longitudinal approach with repeated VAS measurements at six procedural stages. Our baseline-adjusted pain scores model was able to evaluate not only whether music reduced pain, but also how its effect varied throughout the procedure. Moreover, we adjusted for other clinical variables and accounted for within-subject correlation, offering a more comprehensive assessment of music’s impact on procedural pain in a statistically adequate sample.

Although we believe this study provides new evidence on a highly relevant topic, it is not devoid of limitations. First, there was an imbalance in PBx technique, with a higher proportion of fusion-guided PBx in the control group. Although this was addressed through covariate adjustment, stratified analyses, and exploratory interaction testing, differences in procedural complexity and duration may still have influenced pain perception. Second, the absence of a formal anxiety assessment using validated psychometric tools or indirect physiological parameters (such as heart rate and blood pressure) represents a major limitation. To partially account for this, we measured VAS at T0—after digito-rectal examination and prior to initiation of the music intervention—and used this value as an individualized baseline. This early pain score served as a proxy of each patient’s intrinsic pain sensitivity, discomfort tolerance, and psychological state, including anxiety. Future studies should incorporate validated psychometric instruments, such as the State–Trait Anxiety Inventory (STAI) or the Hospital Anxiety and Depression Scale (HADS), to more precisely quantify baseline and procedural anxiety Third, noise cancellation was not feasible as music was played through an external device, possibly limiting the effect of music in alleviating pain. Fourth, because group allocation was not randomized, we adopted a longitudinal analytical approach with repeated pain measurements, adjusted for baseline pain perception using T0, and applied mixed-effects modeling to account for within-subject correlation and relevant clinical covariates. However, these methodological strategies cannot fully substitute for randomization, and residual confounding, as well as potential bias related to patient discontinuation due to pain, cannot be entirely excluded. Lastly, generalizability of these results beyond office-based TP PBx under local anesthesia should be interpreted with caution.

## Conclusion

In this prospective, non-randomized study, listening to music during TP PBx was associated with lower pain perception, particularly during biopsy sampling phases and at discharge. Music intervention may help expanding the use of TP approach as a routinary office-based procedure, even in the absence of anesthesiologist support.

## Supplementary Information

Below is the link to the electronic supplementary material.

Supplementary Material 1

## Data Availability

The data presented in this study are available on request from the corresponding author. The data are not publicly available.
