# Supplementary material for: Impact of music on pain perception during office-based transperineal prostate biopsy: a prospective non-randomized study
Source: Sci Rep. 2026 Apr 24;16:13339. doi: 10.1038/s41598-026-41323-7 (PMC13106671; doi:10.1038/s41598-026-41323-7)
Supplement: Supplementary file 1 — Supplementary Material 1 [file 41598_2026_41323_MOESM1_ESM.docx]

**Supplementary Table 1**. Clinical and demographical variables of patients who did not complete the procedure and were excluded from the study

| **ID** | **Group of Intervention** | **Age, years** | **PSA,**  **ng/ml** | **Type of Biopsy Scheduled** | **Prostate Volume, cc** | **Reason of withdrawal** |
| --- | --- | --- | --- | --- | --- | --- |
| E1 | Control | 77 | 1.8 | Systematic Only | 70 | Intolerance to Rectal Probe (RB) –Symptomatic Hemorrhoids |
| E2 | Control | 59 | 6.5 | Systematic Only | 67 | RB Intolerance – Symptomatic Hemorrhoids |
| E3 | Music | 51 | 13.2 | Systematic + Targeted | 40 | RB Intolerance – previous rectal surgery |

**Supplementary Table 2** Linear Mixed-Effects model estimating D-VAS, An interaction term was introduced to test the impact of Music Intervention on D-VAS at each timepoints.

| Covariates | Estimate | 95% CI Lower | 95% CI Upper | P-value |
| --- | --- | --- | --- | --- |
| **Age** | 0,007 | -0,022 | 0,035 | 0.7 |
| **T2 vs T1** | 1,17 | 0,69 | 1,66 | **<0.001** |
| **T3 vs T2** | 0,89 | 0,39 | 1,37 | **<0.001** |
| **T4 vs T3** | 0,88 | 0,38 | 1,07 | **<0.001** |
| **T5 vs T4** | -0,87 | -1,35 | -0,38 | **<0.001** |
| **Nr of Biopsy cores** | 0,024 | -0,11 | 0,16 | 0,5 |
| **Target + Systematic PBx vs Syst. only** | -0,22 | -0,85 | 0,40 | 0,7 |
| **T1: Music vs Group** | 0.15 | -0,56 | 0,86 | 0,6 |
| **T2: Music vs Control** | -0,20 | -0,90 | 0,51 | 0,1 |
| **T3: Music vs Control** | -0,53 | -1,23 | 0.18 | 0.1 |
| **T4: Music vs Control** | -1,29 | -2,00 | -0,59 | **<0.001** |
| **T5: Music vs Control** | -0,70 | -1,40 | 0.008 | 0,054 |

**Supplementary Table 3** Model-predicted differential pain scores (D-VAS) by intervention group and procedural stage.

| Timepoints | Intervention Group | Predicted D-VAS | 95% CI Lower | 95% CI Upper |
| --- | --- | --- | --- | --- |
| **T1** | Control  Music | 0,47  0,94 | 0,16  0,60 | 0,79  1,28 |
| **T2** | Control  Music | 1,63  1,69 | 1,31  1,35 | 1,94  2,03 |
| **T3** | Control  Music | 1,54  1,28 | 1,22  0,94 | 1,85  1,62 |
| **T4** | Control  Music | 1,26  0,38 | 0,95  0,044 | 1,57  0,72 |
| **T5** | Control  Music | -0.51  -0.72 | -0,82  -1,06 | -0.19  -0.38 |
